# Textured insoles affect the plantar pressure distribution while elite rowers perform on an indoor rowing machine

**DOI:** 10.1371/journal.pone.0187202

**Published:** 2017-11-02

**Authors:** Taian Vieira, Alberto Botter, Laura Gastaldi, Isabel C. N. Sacco, Francesco Martelli, Claudia Giacomozzi

**Affiliations:** 1 Laboratory for Engineering of Neuromuscular System and Motor Rehabilitation, Department of Electronics and Telecommunication, Politecnico di Torino, Torino, Italy; 2 Department of Mechanical and Aerospace Engineering, Politecnico di Torino, Torino, Italy; 3 Laboratory of Biomechanics of Human Movement and Posture, Department of Physical Therapy, Speech, Occupational Therapy, School of Medicine, University of Sao Paulo, São Paulo, Brazil; 4 Department of Cardiovascular, Dysmetabolic and Aging-Associated Diseases, Italian National Institute of Health, Rome, Italy; University of Illinois at Urbana-Champaign, UNITED STATES

## Abstract

**Introduction:**

During rowing, foot positioning on the foot stretcher is critical to optimise muscle force transmission and boat propulsion. Following the beneficial effects of textured insoles on gait and balance, this study aims at investigating whether passive stimulation of foot mechanoreceptors induced by these insoles may contribute to improving foot loading pattern and symmetry during indoor rowing.

**Methods:**

Eleven elite rowers were assessed during controlled training on a standard rowing machine while wearing control, low-density or high-density textured insoles. Plantar pressure and knee and trunk kinematics were measured; performance data were recorded from the machine. Insole effect on kinematic parameters, peak and average values of foot force, contact area and position of centre of pressure was assessed with ANOVA and Bonferroni correction for pair-wise comparisons.

**Results:**

A main effect was observed for force and contact area, with the high-density insoles providing greatest values (P<0.035). No interaction was observed between side and insole (P>0.190), even though symmetry was higher with high-density insoles. Kinematics (P = 0.800) and rowing performance were not affected by insole type; a consistent though not statistically significant increase in mean travelled distance was observed for denser insoles (P>0.21).

**Conclusion:**

The high-density textured insoles affected foot loading distribution during indoor rowing. Rowers applied greater foot force and over a greater foot stretcher area with the high-density than the low-density and control insoles. These findings and the methodology applied may be relevant for the understanding and monitoring of rowing performance.

## Introduction

Powerful muscles and smooth technique contribute markedly to the overall, rowing performance [1]. Small variations in the timing and amplitude of body inter-segmental movements, both within and between rowers, have been suggested indeed to impact negatively on boat velocity [2–4]. Biomechanical and anthropometric determinants of elite rowing performance are also currently under investigation [5–7]. While the markedly high joint contact forces [8] motivate studies on the potential causes of injuries in rowing [9–12], reports specifically aimed at identifying key procedures to safely improve rowing performance are incipient. Acoustic feedback on boat acceleration [13], minimisation of rowers vertical movement [14] and specific crew arrangements [15], for example, have been proposed to affect performance. Interventions aimed at increasing rowing performance by acting on the foot-foot stretcher contact, where propulsive forces are applied to the boat, were though not found in the literature. It is through the foot stretcher that rowers are able to transmit muscle forces to the oar handle and therefore move the boat [16].

Passive stimulation of the mechanoreceptors in the foot may assist rowers in better sensing the foot stretcher and therefore in optimising performance. By distributing nodules throughout plantar insoles, local differences in pressure may indeed be better sensed by the foot mechanoreceptors [17]. Beneficial effects resulting from the use of these insoles, often referred to as textured insoles, have been reported for different populations and circumstances [18–22]. Different older populations, including for example healthy subjects and patients with different clinical conditions, showed improved balance and gait while wearing textured insoles [23–25]. Improvements have been shown to persist weeks after the use of insoles [23,26] and seem to affect differently the left and right sides [27]. The positive effects of textured insoles on athletic performance are less though equally well documented. Soccer and netball players were indeed observed to better discriminate ankle movements when wearing textured insoles [18,26,27]; such a better discrimination ability may assist foot positioning during e.g. landing, improving performance and minimising the risk of injuries. Collectively, these pieces of evidence suggest the effect of textured insoles generalises to a number of circumstances. It is therefore possible the enhanced sensory feedback provided by textured insoles may assist rowers in better distributing force over the foot stretcher, a currently unexplored issue to our knowledge.

In this study we thus investigate whether passive cutaneous stimulation of foot mechanoreceptors may affect the plantar pressure distribution during indoor rowing. We specifically ask: do the contact area and the force applied by both feet to the foot stretchers increase when elite rowers perform on a rowing machine while wearing texture insoles? If stimulation of cutaneous receptors leads to better sensing the contact surface [17,26], we therefore expect such insoles to assist rowers in pushing against a greater foot stretcher area and thus possibly increasing the total foot force. Left-right asymmetry in plantar pressure distribution has been also investigated in the present study since it may be an additional determinant of performance, as well as of musculoskeletal integrity, during rowing. In general, negative correlation between asymmetry and indoor rowing performance has been reported by Longman et al. [4]. Specifically concerning the foot sole, side differences in foot stretcher forces may lead to greater force application on one blade during sculling, resulting in boat yawing and therefore greater drag forces [3,16]. Moreover, asymmetrical foot forces may demand a differential loading of left-right muscles to optimise force transmission to the oar handle, possibly contributing to the stress injuries or back pain [11,12]. To our knowledge this is the first study to systematically report the distribution of foot pressure during rowing, of potentially marked relevance for the understanding and monitoring of rowing performance.

## Materials and methods

### Participants

Eleven (4 females/7 males), elite rowers (18–23 years; 1.70–1.85 m; 58–90 kg) volunteered to participate in this experiment after providing written, informed consent. All participants (also mentioned in the following as either rowers or athletes) have been engaged in competitive rowing for at least four years. One athlete received the gold medal in the 2012 World Rowing Championships (participant 1) and all were gold medallists in national, rowing competitions. Six athletes were starboard rowers and two compete exclusively on sculls. According to the routine medical screening they receive by the National Team Medical Staff, participants did not report any musculoskeletal or foot-related disorders at the occasion of experiments. Experimental procedures conformed to the *Declaration of Helsinki* and were approved by the Institutional Ethics Committee of Politecnico di Torino, Italy.

### Insoles

**Fig 1:**
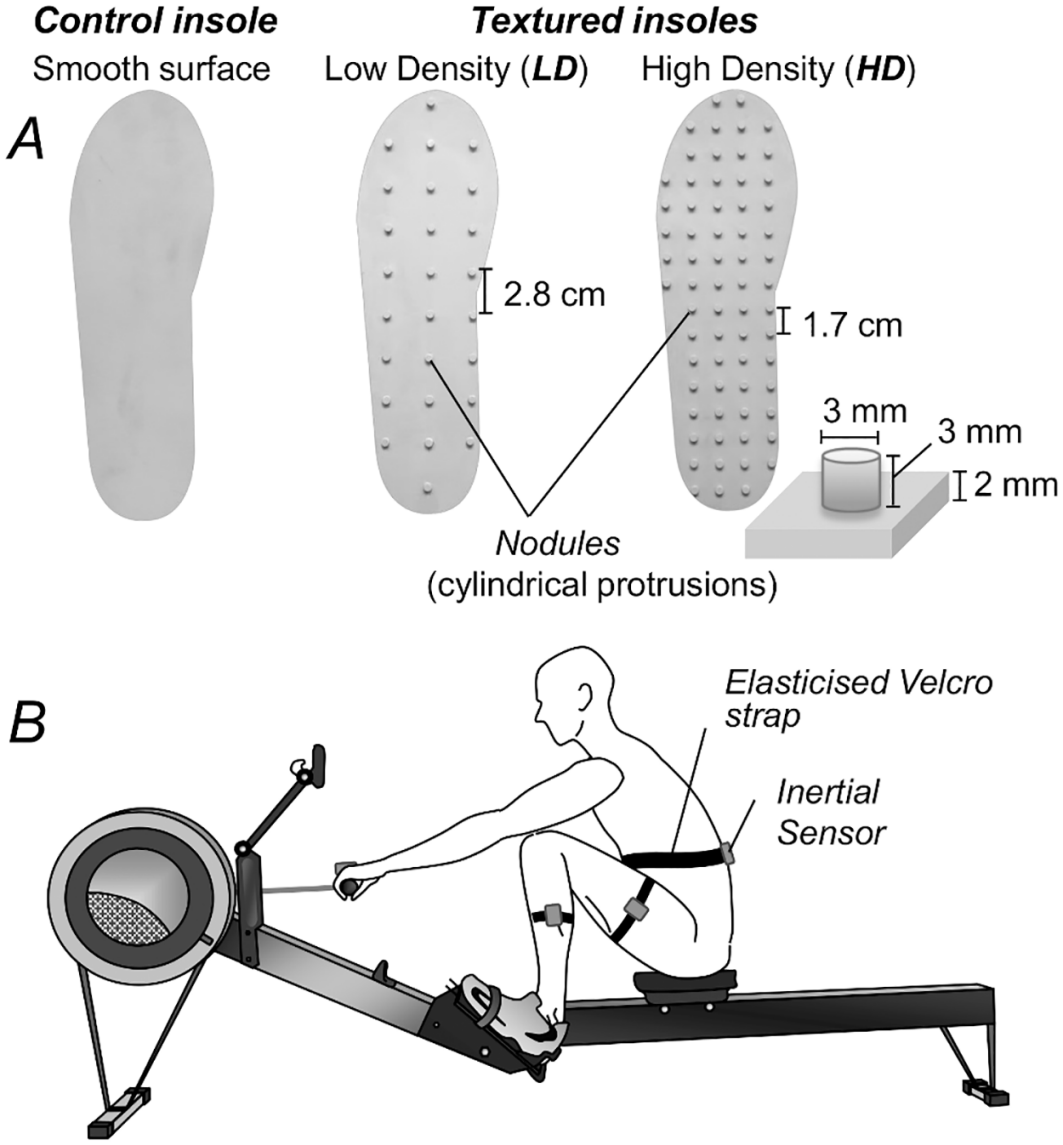
Experimental setup. (A) Insoles for the stimulation of cutaneous receptors (from left to right: control insoles, low-density (LD) insoles, linear spacing between nodules: 2.8cm; high-density (HD) insoles, linear spacing between nodules: 1.7cm. (B) Inertial measurement units (IMUs) arrangement.

Textured insoles have been prepared to passively stimulate feet glabrous mechanoreceptors, according to the literature [18,24,25], by distributing 3 mm height nodules over a medical, rapid-prototyping material (bio-compatible photopolymer MED610; Stratasys, Eden Prairie, MN, United States). Three different insoles were prepared with the same material, depending on the number of nodules per mask (Fig 1A): insoles without nodules (Control insole), with nodules having a relatively high (HD insole) and a relatively low (LD insole) density. Nodules were arranged square over the polymer mask, with 1.7 cm and 2.8 cm distance between adjacent nodules for the HD and LD insoles, respectively. This arrangement was thought to ensure there would be at least one nodule per receptor field in the plantar surface [28]. The number of nodules, depending on the insole size, ranged between 48 and 66 for the HD insoles and between 18 and 24 for the LD insoles. The total thickness, both for the control and textured insoles, amounted to 5 mm (mask and nodes).

### Experimental protocol

Participants were initially instructed to warm up on a rowing machine (Concept II model E, Morrisville, USA) for 5 min. They were asked to self-select and adjust the drag factor of the rowing machine during the warm up session though not during experiments. After that, and based on rolling starts, nine series of at least 30 consecutive strokes each were applied, one for each combination of stroke rate (18, 24 and 32 strokes/min; [11]) and insole (control, LD and HD). Test duration was set at the rowing machine display; 100s, 80s and 60s durations were respectively set for 18, 24 and 32 strokes/min, ensuring the recording of at least 30 strokes per trial. Through the rowing machine monitor, participants were provided with visual feedback on the stroke rate and on the time taken to cover 500 m for individual strokes. Our elite athletes were instructed to maintain the same rate from the beginning of each trial and to minimise the time taken to cover a 500 m distance, i.e. the standard performance metric used by rowers during indoor training. Trials were applied at random order with at least 5 min intervals in-between, to allow athletes to recover and to practice with each of the three insoles prior to starting data collection. Upon completion of the measurement session, participants were asked to indicate whether they felt comfortable or not rowing with each of the three insoles.

### Instrumentation

Pressure data were sampled with the Pedar-X system (Novel_GmbH_, Munich, Germany) at 50 Hz. The accuracy–higher than 5%—of the exploited capacitive technology [29,30], and the reliability of the specific arrangement of the sensor technology in this flexible, in-shoe measurement system (ICC>0.80 for total foot and all variables [31]) render the Pedar system suitable for the accurate pressure measurement this study relies on. For each foot, the instrumented insole—2mm thick and made of 99 calibrated capacitive sensors—was placed in-between the textured insole and the shoe, after removing the standard shoe insole (i.e. the insole that comes with the shoe). Capacitive sensors, once individually calibrated in factory, do not require further calibration before measurements; the only recommended procedure before starting acquisition with the Pedar insoles is the zeroing procedure aimed at removing any possible pre-loading offset. Within this study, zeroing was correctly implemented for each participant at any replacement of textured insoles. Prior to measurements, assessment trials had been conducted to exclude any possible sources of interference between the Pedar-X and the textured insoles. Moreover, at this preliminary stage, none of the volunteers complained about potential differences in cushioning between the insoles used during experiments (textured and instrumented insoles) and the standard insoles they typically wear during training.

Acceleration of the rowing machine handle was measured with an inertial measurement unit (IMU) (MTx, XSens, Enschede, the Netherlands; angular resolution 0.05°; repeatability 0.2°; dynamic accuracy 2° RMS). The sensor was secured tightly to the machine handle to minimise any relative movements between the sensor and the handle. Kinematic data of the left side were acquired with three additional IMUs of the same IMU system–whose accuracy had been proved to be adequate for the study purposes [32] –to monitor the range of knee and trunk flexion-extension. IMUs were secured laterally to the leg and thigh and centrally to the lumbar spine [11,33]. Acceleration and kinematic data were sampled at 50 Hz (output frequency) with a 12 bit A/D converter and offline synchronised with foot pressure data, via an external trigger pulse (TTL signal) provided by the Pedar-X system. A schematic view of the measurement arrangement is provided in Fig 1B.

### Quantifying rowing cycles and pressure parameters

Handle acceleration was considered for the identification of individual rowing cycles. A single rowing cycle is composed of the recovery and drive phases [3]. During recovery, rowers move the handle of the oar (or rowing machine) forward with a sequential, proximal-distal joint movement; shoulder flexion and elbow extension followed by trunk flexion and then hip and knee flexion. The drive phase commences once rowers reach their maximal forward position (catch instant). During the drive phase, the handle is moved backward with a coordinated, distal-proximal joint movement; hip and knee extension followed by trunk extension and then shoulder extension and elbow flexion. The drive phase ends (recovery phase starts) when the handle is brought to rest, roughly at the chest height (finish instants). Catch and finish instants, which define the transition between rowing phases, were defined as peak instants in handle acceleration data [11]. Peak instants correspond to zero crossings in handle velocity and therefore posit the onset of recovery (finish instants: positive peaks) and drive phases (catch instants: negative peaks; cf. Fig 1 in [11]). Peaks were identified with a custom Matlab script (The MathWorks Inc., Natick, Massachusetts, USA). The duration of recovery and drive phases was then computed for each insole and stroke rate. Finish and catch instants were finally considered to segment foot pressure and kinematic data into individual rowing cycles.

**Fig 2:**
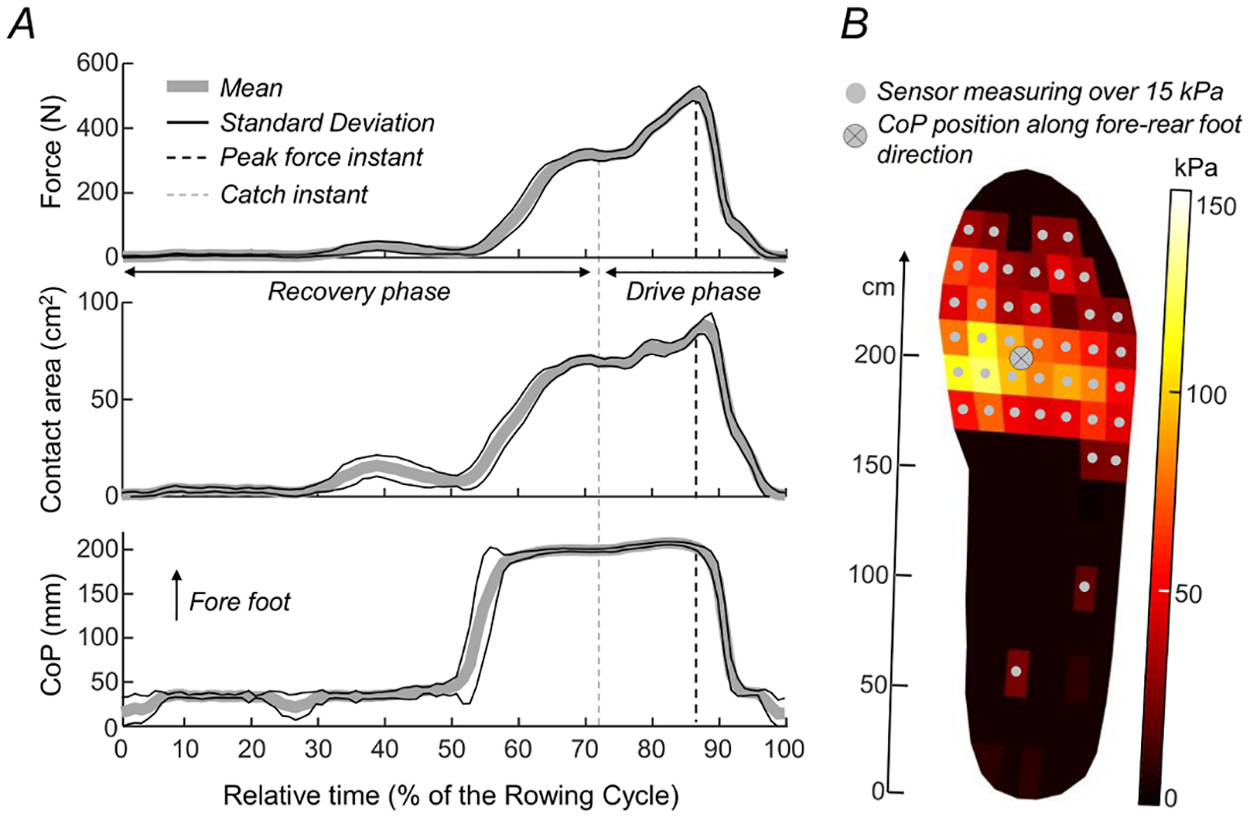
Quantification of foot loading distribution during rowing. (A) Force, contact area and CoP computed for the instant of peak force as well as over the whole drive phase. Data are referred to the right side of participant 6 (male, 18 years, 84 kg, 1.85 m) while rowing at 18 strokes/min with the control insoles. Profiles have been averaged over the 30 central rowing cycles of the trial (mean and standard deviation are respectively shown with continuous and dashed lines). Vertical dashed lines indicate the instant of peak force (black) and the catch instant (grey). (B) Pressure values detected by each of the 99 sensors of the instrumented insole (darker intensities indicate lower pressure values). Circles denote the sensors which pressure value was greater than the 15 kPa threshold, defining the absence of foot contact, whereas the crossed circle indicates the coordinate of pressure distribution along the foot fore-rear direction.

Key variables characterising the rowers’ interaction with the foot stretchers were quantified from the pressure distribution. For each foot we calculated the perpendicular component of the lumped, foot stretcher force vector, the centre of pressure position (CoP) in the rearfoot-forefoot direction, and the contact area (Fig 2A). For each time sample, foot force was defined as the spatial integral of the pressure data whereas CoP was computed as the average of the sensors’ sagittal coordinates weighted by the pressure data. The contact area was defined by summing, for each time sample, the area of sensors providing pressure figures over the 15kPa threshold. Foot force, CoP and contact area time profiles were partitioned into individual rowing cycles and then averaged (Fig 2B). From these average profiles we computed the instant of peak force to test whether rowers pushed maximally at the same relative instant with the textured insoles. Force, CoP and contact area at the peak instant were considered to quantify how strongly and where on the foot stretcher rowers pushed at the instant of maximal force. Finally, during the drive phase, the average force, CoP and contact area profiles were calculated to provide a general indication on how pressure distribution changed with cutaneous stimulation. Variables were quantified for both feet to test for whether the textured insoles could attenuate potential asymmetries in foot pressure during rowing.

Trunk and knee kinematics were computed from the inertial sensors data. Orientation matrix data were converted to Euler angles, which were then considered to quantify changes in trunk and knee angles in the sagittal plane. The range of flexion and extension motion throughout the rowing cycle was considered to assess the effect of insoles on trunk and knee kinematics.

### Statistics

After ensuring the homogeneity of variance (Levene’s test; W values >0.24 for all cases) and the data Gaussian distribution (Shapiro-Wilk statistics; *P*>0.15 for all cases), parametric tests were applied to compare foot pressure data for the different insoles and stroke rates. Multi-factorial ANOVA was applied, with insoles as repeated measures (2 sides x 3 stroke rates x 3 insoles). Bonferroni correction was considered for post-hoc analysis. Differences in the duration of rowing cycles and the effect of textured insoles on kinematics, distance travelled and mean power were tested with two-way ANOVA (3 stroke rates x 3 insoles), with insoles as repeated measures. Based on the error variance and the variance associated with the insoles, we estimated the effect size to range from 31% (contact area at peak force) to 61% (average force during drive; [34] Thirty rowing cycles ensured a high/moderate (range: 46–91%) statistical power [34].

## Results

None of the participants tested complained about rowing with the textured insoles. Five of them reported to row most comfortably with the control insole whereas five and only one respectively preferred rowing with the HD and LD insoles.

All athletes completed all trials at a consistent intra-trial stroke rate (coefficient of variation 0.01–0.04). Inter-trial consistency was also found among the athletes at corresponding stroke rate. The average duration of rowing cycles decreased from 3.1(SD: 0.2)s to 2.4(0.1)s and then to 1.9(0.1)s as the stroke rate respectively increased from 18 to 24 and then to 32 strokes/min (ANOVA main effect; *P*<0.001; *n* = 99; 3 cadences x 11 participants x 3 stroke rates). Such decrease was mostly due to shorter recovery than drive phases [35]; in relation to the rowing cycles, recovery and drive phases lasted respectively 71.6(2.8)% and 28.4(2.8)% at 18 strokes/min, 67.1 (4.4)% and 32.9 (4.4)% at 24 strokes/min and 59.9 (2.4)% and 40.1 (2.4)% at 32 strokes/min. Duration values were averaged across insoles. Except for the duration of rowing recovery phases, stroke rate did not affect force, contact area, CoP and the timing of peak force (ANOVA main effect; *P*>0.23; *n* = 198). For this reason, plantar pressure data were assessed after collapsing the three stroke rates.

**Fig 3:**
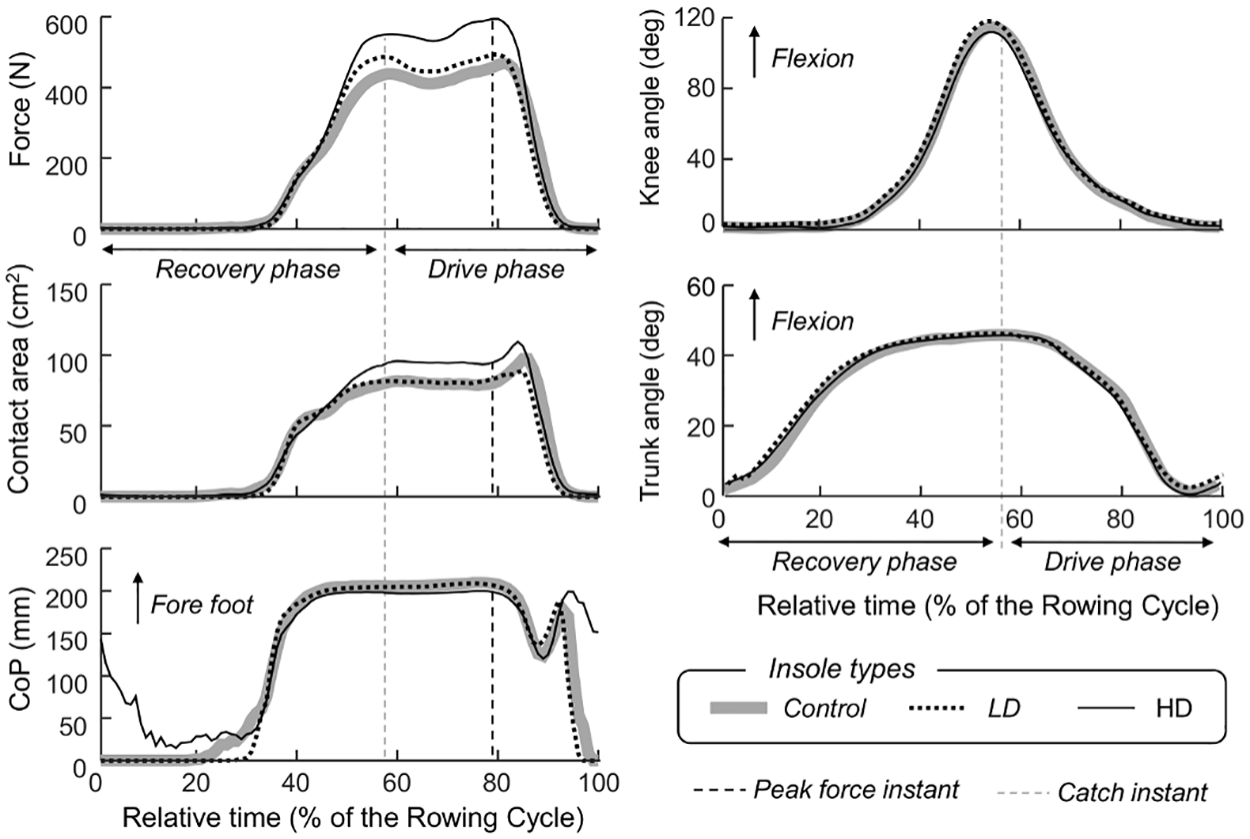
Effect of cutaneous stimulation on foot loading distribution and knee and trunk flexion-extension movements. Representative graphs referred to the left side of participant 6 (male, 18 years, 84 kg, 1.85 m, 32 strokes/min). With the HD insoles, his force and contact area values at peak force were about 21% and 9% greater than those observed for the LD insoles, and 28% and 14% greater than those observed for the control insoles. Similar effect was found for the HD insoles with respect to the whole drive phase: +22% and +14% of force and contact area with respect to LD insoles, +32% and +17% with respect to control insoles. For each insole and parameter, profiles have been averaged over the 30 central rowing cycles of each trial. Data for different insoles are presented with different traces (Control: thick grey line; LD: thin, black dashed line; HD: thin, black line). Vertical black dashed lines indicate the instant of peak force; vertical grey dashed lines indicate the catch instant.

The use of textured insoles affected the distribution of plantar pressure during indoor rowing. Clarifying, representative graphs are reported in Fig 3.

**Fig 4:**
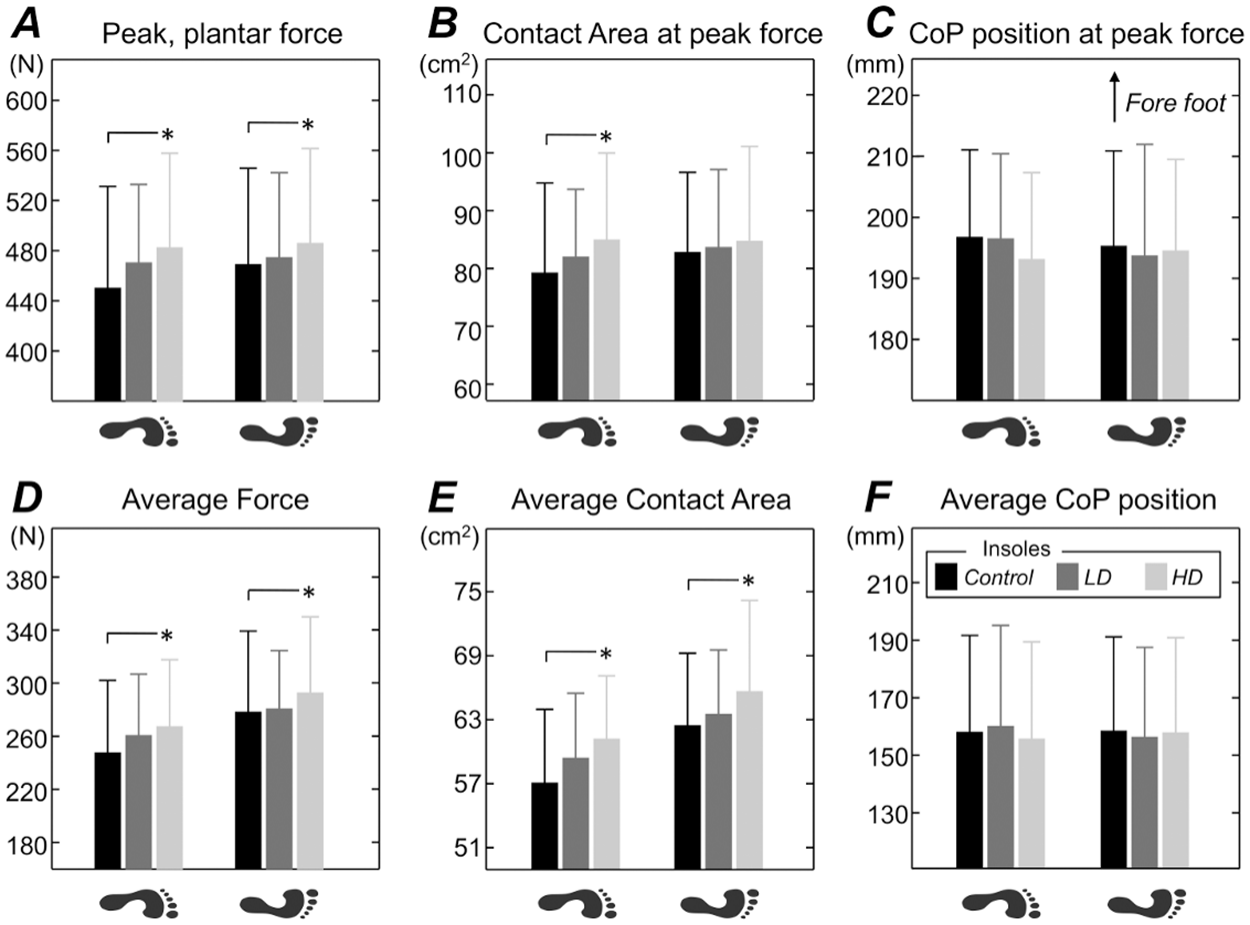
Changes in foot loading with cutaneous stimulation during rowing. Mean and standard deviation (whiskers) values are shown for (A) the peak force, (B) contact area and (C) the fore-rear CoP position at the instant of peak force. Group data averaged over the drive phase are shown in panels (D), (E) and (F) respectively. Different insoles are represented with different grey intensities (black: Control; dark grey: LD insole; light grey: HD insole), separately for the left and right foot. Asterisks denote statistical significance for pair-wise comparisons with Bonferroni correction at *P*<0.05.

When considering all participants, a main effect of insole type on total force and contact area was observed (ANOVA; *P*<0.021; *n* = 66; 11 participants x 3 insoles x 2 feet). The HD insoles provided significantly greater force and contact area at peak force and over the whole drive phase than the *control* insoles (ANOVA Bonferroni correction for pair-wise comparisons; *P*<0.035; Fig 4A, 4B, 4D and 4E). Force and contact area were consistently though not statistically greater for LD than *control* insoles. No interaction was found between side and insole type (ANOVA; P>0.19; n = 66); side-differences in the mean value of force and contact area reduced from ~10% with control to ~6% with HD insoles (Fig 4A and 4B) but they were not significant. No main and interaction effects of side and insole type were found on CoP (ANOVA; *P*>0.22; *n* = 66; Fig 4C and 4F).

Overall power measured by the rowing machine and body kinematics were not affected by the insoles. The mean distance travelled with HD insoles was from 4 to 12 m longer than that travelled with control insoles (Table 1), but not statistically significant (ANOVA main and interaction effect; P>0.21; n = 99; 3 stroke rates x 11 participants x 3 insoles). In general, power slightly increased but it was not significantly greater with the insoles with more nodules (Table 1). Finally, the range of trunk (mean value; SD: 60;8°) and knee (148;17°) motion observed for control insoles was not different (ANOVA main effect; *P* = 0.8; *n* = 33) from that observed for the other insoles (LD: trunk and knee; 59;9°, 148;13°; HD: 60;7°, 153;14°).

**Table 1:** Average power and distance travelled for each of the three stroke rates considered (no main effect for insole type observed, P>0.21 for both parameters).

Stroke rate (stroke/min)	Test Duration *(s)*	Average power (watts)			Distance travelled (m)		
		*Control*	*LD*	*HD*	*Control*	*LD*	*HD*
18	100	219 (51)	219 (54)	222 (56)	423 (34)	422 (39)	428 (47)
24	80	262 (66)	271 (61)	269 (66)	360 (11)	362 (30)	372 (30)
32	60	321 (74)	331 (82)	332 (82)	291 (26)	294 (24)	295 (24)

Legend. Mean (standard deviation) values are reported. See Fig 1 for indications on the Control, LD and HD insoles.

## Discussion

The effect of textured insoles on the plantar pressure distribution was assessed while elite rowers performed on a rowing machine. We hypothesised the stimulation of cutaneous mechanoreceptors, mediated by textured insoles (Fig 1), would lead to greater foot forces, distributed over a larger area and more symmetrically between limbs. From eleven elite rowers, our results show that when compared to control, glabrous insoles, rowing with textured insoles with a high density of nodules resulted in (Figs 3 and 4) greater foot force and larger contact area between the foot and the foot stretcher. Left-right differences in foot force and contact area slightly decreased, but the reduction was not statistically significant. Collectively, these results suggest the textured insoles may potentially assist rowers in optimising foot stretcher forces during rowing, as discussed below. The whole methodology applied for data acquisition and processing, in addition to our key results, resulted feasible and reliable, and may be relevant for the understanding and monitoring of rowing performance.

### Effect of textured insoles on plantar pressure distribution during rowing

The textured insoles affected the distribution of plantar pressure during rowing. When rowing with textured material inserted into their athletic shoes, participants could develop greater forces than when rowing pushing on a smooth surface (Figs 3 and 4). In agreement with previous reports [17,20], such effect was however statistically significant only for the insoles with more nodules (HD insole; Fig 1). Watanabe and Okubo [17], for example, observed the number of neuronal discharges in the tibial nerve was greater for insoles with a greater number of cutaneous, stimulation points. It is possible the HD insole sensitised a greater number of mechanoreceptors [28], thereby increasing sensory feedback on the spacing and orientation of the texture pattern and consequently on the efferent responses produced by the lower limbs over the foot stretcher. Differently from the hand [36], the position of receptive fields is randomly distributed throughout the plantar surface [28]. Such a wide dispersion of receptive fields may serve well for sensing fine changes in plantar pressure distribution, both within and between strokes. The effect of HD insoles was evident both at the instant of peak force and during the whole drive phase (Figs 3 and 4), when rowers push strongly against the foot stretcher through vigorous extension of their legs, trunk and arms. Even though the values of peak force reported here are within the documented range of foot stretcher peak forces (300-650N; [1,3,8]), figures on the mean force across drive as well as on foot contact area and CoP (cf. Fig 2) during rowing were not identified in the literature. Here we show rowers seem to use a small area (~40%), in relation to the average size (~165cm^2^) of their feet, to push against the foot stretcher. And they push predominantly with their forefoot (Figs 2B, 4C and 4F). Our results further show the variations in total force (Fig 4A and 4D) with insole type were accompanied by proportional variations in foot contact area (Fig 4B and 4E). This indicates the textured insoles resulted in greater forces being applied over greater foot stretcher area.

An alternative competing explanation for the altered plantar pressure distribution could have been discomfort. Textured insoles have been used indeed to induce discomfort and therefore to improved kinematics. Aruin and Kanekar [21] used textured insoles alternatively on a single foot to induce discomfort; similarly, Ritchie and colleagues [19] argued the lower ankle pronation they observed during gait could have been partly due to an individual attempt to minimise discomfort resulting from the insole nodules in the midfoot. We however believe discomfort unlikely explains results presented in Figs 2 and 4. If for example participants had increased the foot contact area in an attempt to reduce pressure under individual nodules, greater contact area would have been expected for LD insoles; condition of maximal pressure under individual nodules. Moreover, major, kinematic changes presumably resulting from discomfort were not observed and none of the study volunteers reported discomfort with the insoles upon the end of experiments.

### The potential benefits of textured insoles in rowing

Textured insoles have been used in different fields with the twofold aim of improving performance and preventing injuries [20,23]. Performance is typically conceived in terms of better discrimination of foot orientation in space [26], reduced postural sways [24,25] and reduced gait variability [20]. Improvements in such performance variables may in turn reduce the risk of injuries, as more accurate information about foot position in space and better balance and gait skills may assist people in safely interacting with surroundings (e.g. successful obstacle clearance). In the present study, better performance of the indoor rowing athletes was associated with improved foot loading on an increased plantar pressure distribution surface (Fig 2). The increased foot forces observed in response to the use of HD insoles (Fig 4) is likely associated with greater forces applied to the machine handle, possibly explaining the non-significant though consistently greater average power measured for the denser insoles (Table 1). Given the duration of rowing cycles did not change across insoles, the slight increase in the measured power could be alternatively explained by greater stroke lengths. This possibility seems however unlikely as cutaneous stimulation did not affect the range of knee and trunk motion in the sagittal plane. Notwithstanding the potential sources accounting for increased power, our results suggest athletes could optimise the application of force to the foot stretcher when provided with enhanced sensory feedback of plantar pressure.

A note should be made here on the relevance of the statistically small changes with foot insole reported in Table 1, both for power and distance travelled. Overall power depends on the force applied to the machine handle, which in turn depends on inter-segmental forces developed in response to the foot stretcher reaction forces. It is therefore possible that the increased foot force observed for denser insoles was not sufficiently high for the overall power and distance travelled to increase significantly. Nevertheless, the consistently longer, and likely relevant, distance travelled with the insoles with more nodules suggests the increased foot force may contribute to improving performance. In fact, considering roughly 200 strokes take place in a 2000 m race, small changes in power may represent major changes in general performance. Indeed, when considering the average distance travelled for the different trials, participants reached distances from 3 to 19 m longer when rowing with the HD rather than the *control* insole (Table 1). In relation to the latter, and considering an average 4 m/s boat speed [3,13], we estimate our participants would have travelled equal distances by a shorter duration, from 0.75s to 4.75s, with the HD insole. In agreement with this view, Smith and Hopkins [37] observed minor increases in boat speed (~0.5%) may impact crucially in race times. While the association between foot stretcher forces and performance during indoor rowing remains the subject of future investigations, our results show passive stimulation of foot sole does lead to application of greater forces to the foot stretcher.

Left-right asymmetry in plantar pressure distribution may be an additional determinant of performance, as well as of musculoskeletal integrity, during rowing. Among other circumstances, both for novice and elite rowers, asymmetries have been reported in muscle activation [11], oar forces [38], joint kinematics [39] and, most importantly, in foot stretcher forces [6,39]. These side differences may be detrimental to rowing performance, as suggested by the negative correlation between asymmetry and indoor rowing performance reported by Longman et al. [4]. Specifically concerning the foot sole, side differences in foot stretcher forces may lead to greater force application on one blade during sculling, resulting in boat yawing and therefore greater drag forces [3,16]. Moreover, asymmetrical foot forces may demand a differential loading of left-right muscles to optimise force transmission to the oar handle, possibly contributing to the stress injuries and to the frequent episodes of back pain reported for rowers [11,12,40,41]. Even though the left-right differences observed in foot force for the *control* insole did not reach statistical significance (cf. black bars in Fig 4), they were well in agreement with mean asymmetry values (~10%) reported by others for foot force [6,39]. Strikingly, our results suggest there is a tendency though for denser, textured insoles to attenuate such asymmetry (cf. differences between feet for force and area values shown in Fig 4); side differences in foot force and foot contact area, both as peak and average values, reduced by ~4% (effect size greater than 51% [34]) when comparing HD and control insoles. Additionally, it is worth noting that two out of the 11 subjects tested in our study competed exclusively on sculls; it is thus possible that our results have underestimated the effect of insoles on the reduction of side-differences in foot pressure. Regardless of the causes chiefly accounting for asymmetric rowing [1], and considering hundreds of strokes are performed during competitions and regular training sessions, it seems worth to devise further investigations aimed at clarifying the potential for textured insoles to minimise side differences during rowing.

Although we understand on-water assessment would provide a more comprehensive view of the effect of textured insoles on rowing performance, we limited our investigation to indoor rowing. Two reasons motivated our decision. First, during indoor rowing we could control for the potential effect of confounding factors on the plantar pressure distribution, such as boat roll movements and asymmetric hand movements resulting from oar handles overlapping in sculls. Second, plantar pressure and joint kinematics could not be measured on-water at the time of the assessment without peculiar hardware adaptation. Given rowing simulators seem to reproduce well the lower limb kinematics observed on water [1], one may speculate there is a potential for the effect of textured insoles observed here to apply for on-water rowing as well. However, the above should not be conceived to straight predict on-water performance; rather, they may pose the grounds for devising future protocols aimed at assessing directly the effect of insoles on on-water, rowing performance.

### Comparison with existing literature

It is worth mentioning no previous accounts on the use of textured insoles and on the plantar pressure distribution during rowing were found. Direct comparisons between present and previous results are therefore not possible. Differently from previous studies, here we assessed the effect of textured insoles directly on the plantar pressure distribution, at the interface mediating the responses to stimuli provided by the textured material. Insole effect was assessed during rowing because of the pivotal role foot forces may have on general performance [16,39]. Further, forces developed at the foot stretcher crucially affect boat propulsion and may help discriminating sources of left-right asymmetries among elite rowers [9,12,40].

### Limitations and future perspectives

Inter-individual differences in foot positioning on the foot stretcher and the general posture of participants during rowing could be argued a potential limitation of the study. Even though we did not measure specific, postural variables, we believe any potential variability between subjects would have not affected our current results. Given we considered insoles as repeated measures, the variability due to inter-individual differences is cancelled [42]. We acknowledge though an exhaustive analysis on the direction and magnitude of the lumped, force vector, not possible with the used experimental setup, could reveal additional, relevant features. A potential limitation might also come from the lack of a sham condition. However, while on one side participants were aware of the experimental intervention, attention was paid when informing them on the study to remain as neutral as possible with respect to the positive, negligible or negative effect of each of the three insoles. Finally, different sources other than textured insoles might have been considered for augmented, foot feedback. With vibrotactile devices, for example, the frequency, amplitude and site of plantar stimulation may be well manipulated [43]. In the present study we considered however testing for the effect of textured rather than of vibrotactile insoles, as the former is less expensive and has been used with beneficial remarks in different circumstances [20,23,26]. Most importantly, the textured insoles considered here may be readily used to investigate the effect of cutaneous stimulation on foot stretcher forces and general performance during on-water rowing, once the instrumentation has been adapted for. Relevant research questions about long-term effects of HD insoles during on-water rowing deserve further investigation.
